# FAK Family Kinases in Vascular Diseases

**DOI:** 10.3390/ijms21103630

**Published:** 2020-05-21

**Authors:** James M. Murphy, Kyuho Jeong, Ssang-Taek Steve Lim

**Affiliations:** Department of Biochemistry and Molecular Biology, College of Medicine, University of South Alabama, Mobile, AL 36688, USA; jmurphy@southalabama.edu (J.M.M.); kjeong@southalabama.edu (K.J.)

**Keywords:** FAK, Pyk2, integrin, vascular disease, restenosis, atherosclerosis, heart failure, pulmonary hypertension, aneurysm, thrombosis

## Abstract

In various vascular diseases, extracellular matrix (ECM) and integrin expression are frequently altered, leading to focal adhesion kinase (FAK) or proline-rich tyrosine kinase 2 (Pyk2) activation. In addition to the major roles of FAK and Pyk2 in regulating adhesion dynamics via integrins, recent studies have shown a new role for nuclear FAK in gene regulation in various vascular cells. In particular, FAK primarily localizes within the nuclei of vascular smooth muscle cells (VSMCs) of healthy arteries. However, vessel injury increased FAK localization back to adhesions and elevated FAK activity, leading to VSMC hyperplasia. The study suggested that abnormal FAK or Pyk2 activation in vascular cells may cause pathology in vascular diseases. Here we will review several studies of FAK and Pyk2 associated with integrin signaling in vascular diseases including restenosis, atherosclerosis, heart failure, pulmonary arterial hypertension, aneurysm, and thrombosis. Despite the importance of FAK family kinases in vascular diseases, comprehensive reviews are scarce. Therefore, we summarized animal models involving FAK family kinases in vascular diseases.

## 1. Introduction

Cell–matrix adhesion plays a significant role in maintaining normal tissue structure and in promoting pathological remodeling in many human diseases [1]. Cells interact with the extracellular matrix (ECM) through integrins, a major family of cell adhesion receptors [2]. Integrins form up to 24 αβ heterodimeric receptors comprised of 18 α and 8 β subunits via noncovalent interactions. Specific integrins bind to matching ECM proteins including collagens, laminin, fibronectin, elastin, and vitronectin. Changes in ECM and integrin expression are closely linked to the progression of various vascular diseases including restenosis, atherosclerosis, pulmonary arterial hypertension, heart failure, aneurysm and thrombosis [3,4,5]. Cells of the vessel wall, such as endothelial cells (ECs), vascular smooth muscle cells (VSMCs), macrophages, and platelets, express cell-type specific integrins during healthy and diseased states (reviewed in [4]).

**Figure 1:**
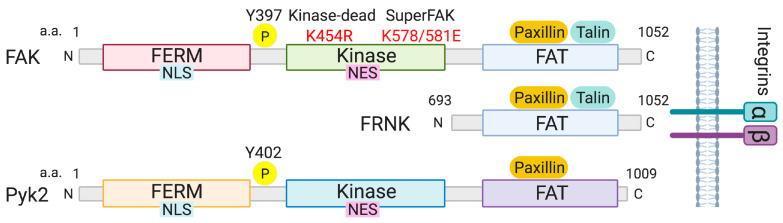
Structure of FAK, FRNK, and Pyk2. The main domains of FAK, FRNK and Pyk2 are shown. FAK and Pyk2 have three major domains: The N-terminal FERM (band 4.1-ezrin-radixin-moesin) domain, the central kinase domain, and the C-terminal focal adhesion-targeting (FAT) domain. FAK and Pyk2 localize to integrin-containing adhesions via their FAT domains. Upon kinase activation, autophosphorylation at tyrosine (Y) 397 FAK and Y402 Pyk2 provides a binding site for Src-homology 2 (SH2) containing proteins. FAK and Pyk2 shuttle between the nucleus and cytosol through a nuclear localization signal (NLS) and nuclear export signal (NES) in their FERM and kinase domains, respectively. FAK kinase-dead (FAK-KD) is a single nucleotide mutation (lysine 454 to arginine) in the kinase domain resulting in loss of kinase activity. SuperFAK contains two mutations (lysines 578/581 to glutamic acids) that increases catalytic activity of FAK. FRNK (FAK-related nonkinase), which comprises only the C-terminal domain of FAK, is an endogenous inhibitor of FAK. Y397: FAK autophosphorylation site. Y402: Pyk2 autophosphorylation site. a.a.: Amino acids. N: N-terminal. C: C-terminal.

**Figure 2:**
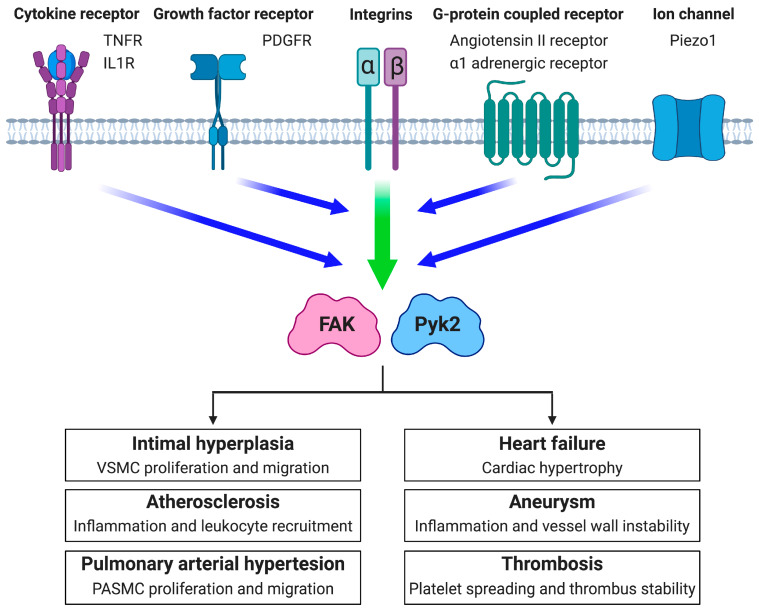
The potential roles of FAK and Pyk2 in vascular diseases. Integrins promote FAK and Pyk2 activation in cooperation with other cell surface proteins including cytokine receptors, growth factor receptors, G-protein coupled receptors, and ion channels. FAK and Pyk2 are major signaling mediators downstream of various signaling molecules during the initiation and continuation of intimal hyperplasia, atherosclerosis, pulmonary arterial hypertension, heart failure, aneurysm, and thrombosis. TNFR: tumor necrosis α receptor. IL1R: Interleukin-1 receptor. PDGFR: platelet-derived growth factor receptor. ⍺-adrenergic receptor: phenylephrine receptor. Piezo1: mechanosensitive ion channel.

As integrins do not have intrinsic kinase activity, ECM signals are transmitted through serine/threonine and tyrosine kinases which mediate cellular adhesion signaling [6,7]. Two key protein tyrosine kinases in integrin signaling are focal adhesion kinase (FAK) and proline-rich tyrosine kinase 2 (Pyk2), which belong to FAK family kinases (Figure 1). Alterations to the ECM that arise in vascular diseases increase corresponding integrin activation, and in turn, lead to elevated FAK or Pyk2 activity (reviewed in [4,8,9]). The ECM–integrin interaction plays an important role not only in promoting cell attachment, but also in facilitating signaling of other cell surface receptors such as growth factors, cytokines, and G-protein-coupled receptors (Figure 2) (reviewed in [10,11]). Signaling through these receptors is often dependent on integrin occupancy as lack of cell attachment has been shown to prevent signaling downstream of the associated receptors [12,13]. Hence, the cooperative signaling through integrin and cell surface receptors enhances FAK or Pyk2 activation to drive vascular disease progression via increased cell migration, proliferation, survival, and altered gene expression (Figure 2).

While the role of FAK family signaling in regulating focal adhesion dynamics via integrins or in further transmitting other surface receptor signaling has been extensively studied, it has been shown that FAK can localize to the nucleus and plays a key role in regulating gene expression by modulating transcription factor stability [14,15,16]. Importantly, our recent study found that FAK is primarily located within the nuclei of VSMCs of healthy arteries and nuclear FAK is inactive. However, vessel injury promotes FAK cytoplasmic translocation where it is activated at integrin adhesion sites and promotes VSMC proliferation [14]. FAK inhibition (by using a pharmacological FAK inhibitor or VSMC-specific FAK kinase-dead (KD) knock-in mouse model) reduces VSMC hyperplasia by forcing FAK localization to the nucleus during vessel injury. This study implicated a potential use of current FAK inhibitors in proliferative vascular diseases. Although a number of exciting animal models involving FAK family kinases within vascular biology have recently been reported, relevant reviews have been scarce. Here, we discuss the role of FAK and Pyk2 in various vascular diseases.

## 2. Structure of FAK Family Kinases

FAK (gene symbol: *PTK2*) and Pyk2 (*PTK2B*) are structurally similar to each other. They are comprised of three main domains: The N-terminal FERM (band 4.1, ezrin, radixin, moesin) domain, the central kinase domain, and the C-terminal focal adhesion targeting (FAT) domain (Figure 1). The fact that the FAK family function not only as kinases but also as kinase-independent scaffolds has increased the complexity of their signaling pathways.

Structural analyses of the FAK FERM domain revealed three distinct subdomains: F1, F2, and F3 lobes [17]. Both FAK and Pyk2 are able to shuttle between the cytosol and nucleus via a nuclear localization sequence (NLS) within the FERM F2 lobe and a nuclear export sequence (NES) in the kinase domain [15,18]. Many nuclear FAK studies have investigated its kinase-independent functions, with most focusing on the regulation of nuclear protein stability [14,15,16,19]. FAK promotes cell survival through recruitment of p53 and its E3 ligase Mdm2 to the FERM F1 and F3 lobes, respectively [15]. This scaffolding role of FAK increases p53 ubiquitination and proteasomal degradation, leading to enhancement of cell proliferation. In addition, we recently showed that nuclear FAK can alter the transcription of genes, such as vascular cell adhesion molecule-1 (VCAM-1) and cyclin D1 in fibroblasts and VSMCs, through FERM-mediated degradation of the GATA4 transcription factor [14,16]. However, it was recently reported that nuclear FAK associated with the RNA polymerase II complex at the vascular endothelial growth factor receptor 2 (VEGFR2) promoter in ECs [20]. Although VEGFR2 transcription was dependent on a nuclear FAK kinase-dependent function, a potential nuclear FAK phosphorylated target was not found [20].

Integrin engagement and co-activation of transmembrane receptors induces rapid autophosphorylation at tyrosine (Y) 397 of FAK and at Y402 of Pyk2 [21] (Figure 1). Src tyrosine kinase binds to these phosphorylated tyrosine residues and further activates FAK and Pyk2 through phosphorylation of a conserved activation loop (Y576/Y577 in FAK and Y579/Y580 in Pyk2) [21,22] (Figure 1). Although Src–FAK complexes may mutually affect downstream signaling, Src has not been rigorously studied in vascular diseases, except in vascular permeability [23,24]. Crystal structure analysis of FAK FERM–kinase revealed that the FAK FERM domain blocks the kinase domain and maintains an auto-inhibited conformation. This auto-inhibited state of FAK is thought to be physically disrupted by the activation of integrins, leading to a conformational shift in FAK and promoting its kinase activation [25,26]. Phosphatidylinositol 4,5-bisphosphate, whose binding sites overlap with the FAK NLS, have been shown to release the FAK FERM–kinase interaction [27,28,29].

The C-terminus of both FAK and Pyk2 contains a FAT domain, which allows them to localize to integrin-containing sites called focal adhesions via paxillin and/or talin binding [30]. The inability of Pyk2 to associate with talin reduces its focal adhesion localization, leading to a more diffuse cytoplasmic or peri-nuclear localization [31]. The FAK C-terminal fragment called “FAK-related nonkinase (FRNK)” was first identified as an endogenous FAK inhibitor within VSMCs [32] (Figure 1). Interestingly, the FAK gene contains an intronic promoter region responsible for the expression of FRNK [33]. FRNK lacks FAK FERM and kinase domains but can still associate with paxillin and talin at focal adhesions, which displaces full-length FAK and inhibits FAK activation [33] (Figure 1).

## 3. FAK Family Kinases in Vascular Diseases

There have been a number of studies on the role of FAK and Pyk2 within vascular cells including VSMCs, ECs, cardiomyocytes (CMs), fibroblasts, macrophages, and platelets (Figure 2). Here, we focus on reviewing what is known about FAK or Pyk2 expression and activity within vascular cells and vascular diseases, with an emphasis on their connection to integrin signaling (Table 1). We will also discuss current FAK and Pyk2 genetic animal models used to study vascular diseases.

### 3.1. Intimal Hyperplasia and Restenosis

Intimal hyperplasia is a type of vascular remodeling that arises from the proliferation and migration of VSMCs into the intima. This process occurs in various pathological conditions (i.e., atherosclerosis and pulmonary arterial hypertension) and during restenosis following clinical procedures such as angioplasty and vein graft [46,47,48,49]. Under healthy conditions, VSMCs are primarily surrounded by basement membrane ECMs such as collagen IV, laminin, and elastin which bind to α1β1, α2β1, and α3β1 integrins (reviewed in [4]). However, in pathological remodeling conditions, the ECM composition changes through the increased secretion of remodeling enzymes, such as matrix metalloproteinases (MMPs) and fibrotic ECM proteins including fibronectin, collagen I, vitronectin, and osteopontin which bind to α5β1 and αvβ3 integrins [4]. Early studies in vitro demonstrated that VSMCs proliferate more rapidly on fibronectin than on laminin [50]. Platelet-derived growth factor (PDGF) can induce VSMC proliferation via ERK MAPK activation in VSMCs plated on either fibronectin or laminin. However, PDGF can only activate FAK in VSMCs plated on fibronectin but not on laminin [50], suggesting that laminin binding integrins (α1β1, α2β1, α3β1) may not lead to PDGF-induced FAK activation. While PDGF was also shown to activate Pyk2 and promote VSMC proliferation through ERK and AKT activation [51], the role of integrins in Pyk2 activation in VSMCs has not been elucidated. Increased expression of α5β1 and αvβ3 integrins were found in VSMCs cultured on fibronectin compared to laminin or Matrigel [34]. This increase in α5β1 and αvβ3 integrins was also associated with increased FAK expression and activity, further supporting an important role for FAK signaling downstream of fibronectin-binding integrins in VSMCs. These studies indicate that basement membrane-binding integrins might maintain low FAK activity upon growth factor signaling and reduce VSMC proliferation and migration.

Differentiated VSMCs of healthy arteries are unique in that they abundantly express the endogenous FAK inhibitor FRNK [32,52,53]. Overexpression of FRNK decreases FAK activity, leading to reduced VSMC proliferation and migration [32]. Mutating L1034S (leucine to serine mutation) on FRNK reduced its localization to focal adhesions by decreasing the FRNK–paxillin interaction [53]. L1034S FRNK failed to inhibit FAK activation in response to angiotensin II in VSMCs when compared to wild-type (WT) FRNK, supporting the role for FRNK displacement of FAK at focal adhesions. Interestingly, FRNK expression is altered depending on the ECM species to which VSMCs are attached. FRNK expression was elevated in VSMCs plated on Matrigel or perlecan (a basement membrane proteoglycan), but was decreased in VSMCs plated on fibronectin [54]. ECM and integrin regulation of FRNK expression inversely correlates with FAK activity (pY397 FAK) within VSMCs.

Increased expression of fibronectin that occurs during intimal hyperplasia leads to increased vascular wall stiffness, which in turn activates FAK to promote VSMC proliferation and migration [55,56,57]. VSMCs plated on stiff hydrogels show elevated FAK activation and increased cyclin D1 expression compared to VSMCs on soft hydrogels, implicating that FAK can mediate mechanosignaling by sensing matrix stiffness [56,57]. The importance of FAK expression in vascular remodeling was examined by using a VSMC-specific Myh11-Cre mouse model to knockout (KO) FAK within VSMCs [55]. Loss of FAK expression in VSMCs significantly blocked wire injury-induced vascular remodeling compared to WT mice [55].

However, the differences between FAK kinase-dependent and -independent functions in VSMC proliferation have not been delineated. A recent study has investigated which of these FAK functions were critical for VSMC intimal hyperplasia by using either a pharmacological inhibitor or a VSMC-specific FAK KD knock-in mouse model during femoral wire injury [14]. Pharmacological or genetic FAK inhibition significantly blocked neointimal hyperplasia by forcing FAK localization to the nucleus and inhibiting its kinase activity when compared to the control mice, suggesting that both reduced FAK activity and increased FAK nuclear localization are important for reducing VSMC proliferation. Interestingly, the majority of FAK appeared to be within the nuclei of VSMCs of healthy femoral arteries and wire injury induced FAK activation and cytoplasmic relocalization [14]. This was the first study demonstrating that FAK may be sequestered inside the nucleus of VSMCs in vivo, which gives a glimpse into a previously unknown role of nuclear FAK. The study further showed that nuclear FAK limited VSMC proliferation by binding to and promoting the proteasomal degradation of GATA4 [14]. Following injury, GATA4 protein expression increased as FAK relocated from the nucleus to the cytoplasm, resulting in VSMC proliferation through direct regulation of cyclin D1 transcription [14]. Forced nuclear FAK localization through the use of a pharmacological FAK inhibitor or in VSMC-specific FAK-KD mice prevented GATA4-mediated cyclin D1 expression and VSMC hyperplasia [14]. These studies help to bridge the knowledge gap between FAK expression and activity in VSMC intimal hyperplasia that was previously unsolved in other studies [56,57]. In addition to promoting cyclin D1 expression, FAK activity has also been linked to the stability of S-phase kinase-associated protein 2 (Skp2), which promotes the degradation of the cell cycle inhibitors p21 and p27 [58]. It has been shown that inhibition of FAK by overexpression of either FRNK or the inactive FAK Y397F (tyrosine to phenylalanine) mutation reduced Skp2 expression and MG-132 (a proteasomal inhibitor) treatment prevented loss of the Skp2 protein. The study suggested that FAK activity may be important for regulating Skp2 protein stability [58]. We have found that nuclear FAK binds Skp2 and promotes Skp2 ubiquitination and proteasomal degradation (unpublished data). Taken together, these studies demonstrate that nuclear FAK suppresses VSMC proliferation by reducing expression of both cell cycle promoters (cyclin D1, Skp2) and increasing expression of cell cycle inhibitors (p21, p27). Although it will need further testing, it is plausible that the high levels of FRNK found in VSMCs may contribute to the increased nuclear FAK and inactive FAK that we observed within VSMCs of healthy arteries.

### 3.2. Atherosclerosis

Atherosclerosis is a chronic inflammatory disease of the vessel wall that results in the excess accumulation of lipids under the endothelium and VSMCs. As atherosclerosis tends to develop in areas of the vasculature that experience disturbed flow (usually in branch or bifurcated points and the inner aortic arch), several studies have investigated how this mechanical stimulation can lead to atherosclerosis progression [59]. The role of integrins in flow-induced signaling of ECs has been elucidated by investigating how different integrin heterodimers affect the EC response to different types of flow [38,41]. In early atherosclerotic lesions, changes in the ECM from collagen to fibronectin induces activation of both αvβ3 and α5β1 integrins [60], both of which lead to FAK activation and subsequent pro-inflammatory molecule expression [38,41]. Flow-mediated activation of integrins triggers activation of FAK and Src, which promote subsequent VEGFR2–Cbl complex formation leading to IKK-NF-κB activation in ECs [61,62]. Treatment with general tyrosine kinase inhibitors (genistein or AG82) reduced flow-induced NF-κB activation and nuclear localization [62], potentially through FAK and Src inhibition. The importance of FAK in flow-mediated signaling was further investigated by using FAK KO mouse aortic ECs [37]. Interestingly, this study found that β1 integrin activating antibodies did not promote transcriptional activation of NF-κB and ICAM-1 expression in FAK KO ECs [37], suggesting that FAK downstream of β1 integrin activation is important for flow-induced inflammation in ECs. It seems that α5β1, but not αvβ3, is required for disturbed flow-induced activation of FAK and NF-κB signaling in ECs [38]. Additionally, both α5β1 and FAK were activated in the inner aortic curvature (under disturbed oscillatory flow), but not in the outer aortic curvature (under linear flow) of low-density lipoprotein receptor (LDLR) KO mice fed a western diet [38]. Crosstalk between the mechanosensitive ion channel Piezo1 with disturbed-flow activation of α5β1 integrin was required for FAK activation and pro-atherogenic inflammatory signaling in ECs [38]. Disturbed flow was also shown to promote the activation of FAK in ECs through increased expression of semaphorin 7A, a transmembrane protein containing an RGD (Arg-Gly-Asp) motif which can serve as an α1β1 ligand [40]. Semaphorin 7A overexpression increased FAK activation and pro-inflammatory molecule expression [40]. On the contrary, integrin αvβ3, but not α5β1, was shown to be important for high shear flow-induced FAK and NF-κB activation [41]. High shear stress is typically found within occluding arteries, suggesting that different integrins are activated at different stages of atherosclerosis [41]. Together, these studies indicate that FAK is a key signaling mediator downstream of various integrins under differential flow conditions in ECs during the initiation and progression of atherosclerotic lesions, making it a potential candidate for the treatment of atherosclerosis.

Low-density lipoproteins (LDLs) that are trapped in the subendothelial layer can undergo several modifications, including becoming oxidized LDL (oxLDL). OxLDL can be endocytosed by several cell types within the vessel wall, such as macrophages, VSMCs, and ECs, which promotes a pro-inflammatory and atherogenic environment [63,64]. ECs more readily induce pro-inflammatory molecule expression in response to oxLDL stimulation when plated on fibronectin compared to basement membrane ECM [39]. This increased inflammatory response was found to be through integrin α5β1-mediated FAK activation [39]. Follow-up studies revealed that FAK activation by oxLDL led to ERK-RSK (ribosomal S6 kinase)-NF-κB activation to promote inflammatory VCAM-1 expression and monocyte recruitment [65]. Interestingly, FAK activity in the ECs of human atherosclerotic lesions is higher when compared to healthy arteries. An EC-specific FAK KD knock-in mouse model from a C57BL/6 background also reduced western diet-induced macrophage recruitment compared to EC FAK WT mice [65].

Atherosclerosis is a chronic inflammatory condition with elevated levels of inflammatory cytokines, such as tumor necrosis factor-α (TNF-α) and interleukin-1β (IL-1β), secreted by activated ECs and macrophages within atherosclerotic lesions [16,66]. Recently, it was shown that dual inhibition of FAK and Pyk2 reduced TNF-α and IL-1β induced pro-inflammatory molecule expression in human ECs [66]. However, inhibition of FAK alone or siRNA knockdown of FAK or Pyk2 only reduced some pro-inflammatory molecules, suggesting that pan-inhibition of FAK family kinases is required to suppress TNF-α and IL-1β signaling in human ECs. By using a carotid ligation model in apolipoprotein E (ApoE) KO mice fed a high fat/high cholesterol (HF/HC) diet, it was shown that FAK activity was important for VCAM-1 expression and macrophage recruitment in vivo [66]. A more recent study showed that ApoE KO and LDLR KO mice fed a HF/HC diet laced with a FAK inhibitor had reduced atherosclerotic lesions and macrophage recruitment, implicating the potential effectiveness of FAK inhibition in treating atherosclerosis [67]. Taken together, these findings demonstrate the important role of FAK in promoting atherosclerosis and inflammation under various stimuli, and that inhibiting FAK activity could reduce atherosclerotic lesions.

### 3.3. Pulmonary Arterial Hypertension

Pulmonary arterial hypertension (PAH) is a pathological form of high blood pressure in the lung due to vascular remodeling of distal pulmonary arteries, leading to hypertrophy of vascular media and intima SMCs, increased arterial pressure, resistance, and plexiform lesion formation. As the lung experiences a variety of mechanical stimulation, studies have focused on the role of the ECM, integrins and their downstream effectors in PAH [68,69]. FAK activation by αvβ3 integrins was shown to be important for pulmonary artery SMC (PASMC) proliferation [35]. Hypoxia triggers PASMC proliferation via increased expression of integrin αvβ3 and FAK activation [35]. Hypoxia also increased αvβ3 integrin binding to osteoprotegerin, a secreted protein upregulated in PAH patients, which in turn led to FAK activation [35]. Knockdown of FAK using siRNA reduced osteoprotegerin-induced PASMC proliferation in vitro. During hypoxic conditions, Pyk2 led to the activation of an ERK-NF-κB-Nox4-H_2_O_2_ pathway that reduced the expression of PPARγ, which in turn increased Pyk2 activation leading to a feed-forward loop that promoted PASMC proliferation [70]. In hypoxia-induced PAH in mice, Pyk2 KO mice showed reduced PASMC proliferation and reduced medial thickness in the lung compared to WT controls [71]. These studies indicate that both FAK and Pyk2 play important roles in the proliferation of PASMCs in hypoxic conditions, which drives PAH disease progression.

It has been shown that PASMCs isolated from PAH patients exhibited higher migratory activity upon PDGF stimulation compared to those from healthy patients [72]. Interestingly, PAH PASMCs express higher levels of the PDGF receptor and showed increased FAK activity. Pharmacological FAK inhibition efficiently reduced PAH PASMC migration as well as prevented downstream pathways including p21-activated kinase, p38, and JNK MAPK signaling, which are known signaling contributors to PAH progression. In a monocrotaline-induced PAH model in rats, treatment with a FAK inhibitor (PF-228) or siRNA against FAK significantly reduced symptoms of PAH [73]. Loss of either FAK activity or expression reduced nuclear localization of STAT3 and active pY705 STAT3, which blocked PASMC migration in a scratch wound assay. These studies showed the potential of FAK inhibitors in reducing PASMC proliferation, migration and survival in a PAH model. More studies are needed to fully elucidate the role of FAK in PAH progression.

### 3.4. Heart Failure

Several vessel narrowing and other systemic diseases that lead to increased blood pressure ultimately result in heart failure as the heart is unable to adequately supply the body with blood containing oxygen and nutrients. These vessel narrowing diseases overload the heart capacity and initially cause hypertrophy of cardiac muscle cells (i.e., CMs). If the underlying causes are not addressed, this increased cardiac hypertrophy will eventually result in cardiac dystrophy and heart failure. FAK and Pyk2 have been shown to play an important role in the progression of heart failure.

As increased blood pressure results in elevated mechanical stress on the heart, early studies investigated the role of β3 integrins in the progression of cardiac hypertrophy. It has been shown that FAK–β3 integrin association is elevated in pressure-overloaded hypertrophic hearts [42]. Increased expression of collagen type III, fibronectin, osteopontin, and β1, α3, and α1 integrin subunits has also been correlated with advancement of cardiac hypertrophy [74,75,76]. In a phenylephrine (PE)-induced hypertrophy model, FAK activation and hypertrophy is dependent on the ECM species to which the CMs are attached. While laminin and fibronectin promoted PE-induced FAK activation and CM hypertrophy, collagen type I and gelatin failed to activate FAK or promote hypertrophy, suggesting that certain ECM–integrin associations are required for hypertrophy [77]. On the contrary, overexpression of FRNK reduced PE-induced FAK activation and blocked hypertrophy, potentially through decreased ERK MAPK activation.

Interestingly, FAK and Pyk2 exhibit differential expression and activation status during the formation of cardiac hypertrophy and progression into heart failure in vivo. While both FAK and Pyk2 showed increased expression and activation in a pressure-overload model of heart failure, elevated Pyk2 expression preceded the development of left ventricular hypertrophy (LVH) [78]. On the other hand, FAK expression was highest during heart failure [78]. FAK expression was also shown to be critical for cardiac hypertrophy in both a CM-specific FAK KO model and mice treated with FAK siRNA [79,80]. CM-specific FAK KO mice was generated by crossing FAK flox/flox mice with MLC2v-Cre mice [81]. CM-specific FAK KO mice attenuated hypertrophy compared to WT mice after four weeks of transverse aortic constriction, suggesting that FAK expression may contribute to the initiation of cardiac hypertrophy [79]. In a related study, it has been shown that siRNA knockdown of FAK was able to prevent and reverse overload-induced LVH in vivo, suggesting that FAK expression is required for the progression of hypertrophy [80]. It is not yet known if FAK activity or cellular localization is important in the progression of cardiac hypertrophy.

Heart failure can lead to an inadequate supply of blood and oxygen to CMs, resulting in a heart attack. From studies that have tried to identify which proteins play a protective role during ischemia and reperfusion, FAK has been identified to play a protective role [82,83]. Using a CM-specific FAK KO model to evaluate the protective role of FAK during ischemia/reperfusion, it was found that mice lacking FAK in CMs had an increased infarct area and increased apoptosis [82]. In another study, a transgenic mouse expressing a super-activatable FAK mutant (K578E/K581E, termed SuperFAK) with increased FAK catalytic activity had a decreased infarct area following ischemia/reperfusion [83], suggesting that FAK activity within CMs is critical for protection against ischemia/reperfusion injury. In contrast to FAK, a recent study showed that Pyk2 activation may be detrimental to CMs following ischemia/reperfusion. Ischemia/reperfusion increased active pY402 Pyk2 in mouse hearts and was associated with increased inhibitory phosphorylation of Y656 of eNOS [84], a known Pyk2 target protein [85]. Mice treated with a dual FAK/Pyk2 inhibitor had decreased infarct areas following ischemia/reperfusion in WT but not eNOS KO mice [84], suggesting that Pyk2 inhibition of eNOS is what drives tissue damage following ischemia/reperfusion. These studies indicate that FAK and Pyk2 may have opposing roles during myocardial infarction; as such, more studies are needed to better understand their roles in heart failure.

### 3.5. Aneurysm

Aneurysm is an excessive enlargement caused by a weakening artery wall, often occurring in the vessels of the abdomen, brain, back of the knee, intestine, or spleen. Alterations to the ECM, such as elastin degradation, and decreased VSMC content are some common characteristics found in aneurysms (reviewed in [86]). Deletion of both integrin α5 and αv in VSMCs using SM22α-Cre led to the formation of large aneurysms within the brachiocephalic artery during embryogenesis [36]. VSMCs isolated from α5/αv integrin KO mice only form nascent adhesions instead of assembling into mature focal adhesions. As a result, α5/αv integrin deficient VSMCs showed decreased active pY397 FAK and reduced phosphorylation of paxillin and p130 Cas. However, a link between FAK activity and the formation of a brachiocephalic aneurysm has not been determined in vivo. In human and mouse abdominal aortic aneurysms (AAAs), increased expression of periostin, an ECM protein interacting with αvβ3 and αvβ5 integrins, was associated with inflammatory cell infiltration and degradation of elastin layers [87]. Rat VSMCs subjected to mechanical strain showed elevated FAK activity, which was reduced by pretreatment with a periostin-neutralizing antibody. Pharmacological FAK inhibition reduced the expression of monocyte chemoattractant protein-1, matrix metalloproteinase-9 (MMP-9), and MMP-2, in human AAA tissue samples ex vivo [87], suggesting that FAK activity in VSMCs may drive sustained inflammation in AAAs. In addition to VSMCs, FAK in macrophages have also been shown to play an important role in the progression of aneurysms. Increased FAK expression and activity were found within human AAA samples when compared to control aortas [88]. Immunostaining revealed that the increase in active FAK was primarily concentrated to the CD68+ macrophage population. FAK activity was required for TNF-α-induced expression of MMP-9 through NF-κB activation in murine macrophages. In a CaCl_2_-induced AAA mouse model, pharmacological FAK inhibition reduced AAA formation through decreased macrophage recruitment and MMP-9 and MMP-2 expression [88]. These studies suggest that FAK inhibitors could be used in patients in which a potential aneurysm has been detected.

### 3.6. Thrombosis

Upon blood vessel injury, platelets adhere to subendothelial ECM proteins and become activated. If a thrombus breaks off from the vessel wall, it can lead to an embolism, which is an obstruction of an artery resulting in myocardial infarction and ischemic stokes. Platelets attach to von Willebrand factor in damaged vessels through αIIbβ3 integrin and the glycoprotein (GP) Ib-IX-V. Upon adhesion and activation, platelets spread out and secrete fibrinogen-containing granules, which also bind αIIbβ3 integrin and promote the adhesion of more platelets at the site of injury [89]. FAK activation in platelets is dependent on both occupancy of αIIbβ3 and a costimulatory molecule such as epinephrine, ADP, or thrombin [43,90]. These co-stimuli increase intracellular calcium and protein kinase C activation, which in turn activates integrin–FAK signaling. The role of FAK in platelets was tested using a megakaryocyte lineage-specific platelet factor 4 (Pf4)-Cre FAK KO mouse model [91]. Deletion of FAK led to prolonged tail bleeding and decreased platelet spreading, suggesting that FAK expression is required for proper platelet function and thrombus stability. However, it seemed that platelet aggregation in FAK KO mice was normal in response to thrombin and ADP [92]. Since both WT and FAK Pf4-Cre KO mice showed similar arterial occlusion times in a FeCl_3_ model of thrombosis, this might have been due to compensatory Pyk2 upregulation and activity [91]. While the study showed that PF-228 (a FAK-specific inhibitor) had no effect on arterial occlusion times, PF-271 (a dual FAK/Pyk2 inhibitor) prevented FeCl_3_-induced arterial occlusion [92]. This finding indicated that both FAK and Pyk2 activity may be important for thrombus formation.

Recent studies showed that Pyk2 has multiple effects on platelet function and plays important roles downstream of both integrins and G-protein coupled receptors (GPCRs). Human platelet attachment to either monomeric type I collagen or GFOGER peptide, a specific ligand for integrin αIIb, activates Pyk2 [45]. Pyk2 activation was also dependent on phospholipase C γ-mediated intracellular calcium release. Pyk2 then promoted the activation of phosphatidylinositol-4,5-bisphosphate 3-kinase β to induce αIIbβ3 inside-out signaling [45]. Further, αIIbβ3 integrin outside-in signaling through fibrinogen activates Pyk2 in platelets [44]. Using Pyk2 KO platelets, they showed that Pyk2 was required for phosphorylation of c-Cbl, an SH2 domain-containing adapter protein. Taken together, these findings demonstrate that FAK and Pyk2 may share distinct and overlapping roles in regulating platelet functions, including platelet production, platelet activation, hemostasis, and thrombosis. The analysis of FAK/Pyk2 double conditional KO mice would certainly help to clarify the possible compensation effect and are needed for a more complete understanding of the regulation of platelet functions.

## 4. Conclusions

Here we have discussed the close association between FAK family kinases and integrin signaling in vascular diseases. As the ECM composition changes during disease progression, the integrin species switch to ones that promote FAK and Pyk2 activation. Increased expression of extracellular signaling molecules such as growth factors, modified lipids, and cytokines further increase FAK and Pyk2 activation in vascular disease through integrin crosstalk with other cell surface receptors (Figure 2). Many vascular cells express both FAK and Pyk2 which may have overlapping roles in particular signaling pathways. In ECs, genetic deletion of FAK has been shown to cause a compensatory upregulation in Pyk2 expression and activity [93], which can make it difficult to distinguish the importance between more complex FAK family kinases. Few FAK or Pyk2 studies have tried to delineate the importance of each one [66,92].

Our recent study demonstrated that FAK is primarily found within the nuclei of VSMCs in healthy arteries and that injury promoted FAK cytoplasmic relocalization and FAK activation in vivo [14]. However, further investigation into how integrins regulate this redistribution of FAK in vascular disease is still needed. Another line of study suggested that mechanical stimulation may regulate FAK localization through integrin signaling. It was demonstrated that stretching of CMs results in FAK nuclear localization in vitro, where FAK binds to the MEF2 transcription factor and regulates CM gene expression [94]. FAK subcellular localization in different vascular cells in vivo and its possible regulation through integrins warrant further investigation and consideration in both healthy and diseased conditions.

Here we described several different genetic animal models used to study the role of FAK or Pyk2 in vascular diseases (Table 2). Most studies described herein made use of tissue-specific Cre lines crossed with FAK flox/flox mice to give rise to tissue-specific FAK deletions. While these studies are useful in evaluating the role of FAK expression during various disease states, we demonstrated that use of a cell-type specific FAK KD knock-in model allowed for the evaluation of FAK kinase-independent functions in VSMCs [14]. For treatment of various vascular diseases, small molecule FAK inhibitors should be tested as a potential treatment option. A big advantage that FAK inhibitors could have over current therapies is that FAK can be delivered systemically without signs of toxicity [95,96,97,98,99]. We hope to see more FAK and Pyk2 research within the vascular field in the future.

## Figures and Tables

**Table 1:** Role of integrins on FAK family signaling in vascular cell types.

Cell Type	Integrin	Function	Reference
Vascular smooth muscle cells	α5β1	Binds fibronectin and promotes FAK activation	[34]
	αvβ3	Binds fibronectin and promotes FAK activation	[34]
		Promotes FAK activation upon binding to osteoprotegerin under hypoxic conditions	[35]
	α5	Dual knockout using SM22α-Cre reduced FAK activity and tyrosine phosphorylation of downstream target proteins	[36]
	αv		
Endothelial cells	α5β1	Promotes flow-induced FAK-mediated NF-κB transcriptional activation	[37]
		Promotes disturbed flow activation of FAK	[38]
		Mediates oxidized LDL activation of FAK	[39]
	α1β1	Increases FAK activation upon binding to semaphorin 7A	[40]
	αvβ3	Promotes flow-induced FAK-mediated NF-κB transcriptional activation	[37]
		Promotes high shear flow-induced FAK expression and inflammatory gene expression	[41]
Cardiomyocytes	β3	Increased association with FAK in pressure-overloaded hypertrophic hearts	[42]
Platelets	αIIbβ3	Activates FAK upon binding fibrinogen in conjunction with costimulatory molecules like ADP, epinephrine, and thrombin	[43]
		Activates Pyk2 upon binding fibrinogen to promote phosphorylation of c-Cbl	[44]
	α2β1	Activates Pyk2 to promote aIIbb3 inside-out signaling	[45]

**Table 2:** Genetic FAK and Pyk2 mouse models to study vascular disease.

Target Cells (Genotype)	Specific Cre or Modification	Result	Reference
Cardiomyocytes (FAK KO)	Nkx2-5 Cre	Worsened ischemia/reperfusion infarct injury	[82]
Cardiomyocytes (FAK KO)	MLC2v Cre	Attenuated hypertrophy	[79]
Cardiomyocyte (SuperFAK transgene)	βMHC promoter drives SuperFAK expression	Protected against ischemia/reperfusion	[83]
Vascular smooth muscle cells (FAK KO)	Myh11 CreERT2	Prevented N-cadherin expression and intimal hyperplasia	[55]
Vascular smooth muscle cells (FAK KD knock-in)	Myh11 CreERT2^T2^	Prevented GATA4-induced cyclin D1 expression and intimal hyperplasia	[14]
Global (FRNK KO)	Deletion of FRNK promoter	SMCs were unable to re-differentiate after artery ligation	[52]
Endothelial cells (FAK KD knock-in)	SCL CreERT2	Reduced macrophage recruitment and VCAM-1 expression in C57BL/6 mice fed high fat diet	[65]
Platelets (FAK KO)	Pf4 Cre	Impaired platelet spreading and increased tail rebleeding	[91,92]
Platelets (Pyk2 global KO)	Deletion of Pyk2 gene	Decreased hypoxia-induced PAH and pulmonary vessel thickening	[71]
		Impaired platelet activation, hemostasis, and thrombosis	[44,45]

## Abbreviations

AAA	abdominal aortic aneurysm
ApoE	apolipoprotein E
CM	cardiomyocyte
EC	endothelial cell
ECM	extracellular matrix
FAK	focal adhesion kinase
FAT	focal adhesion targeting
FERM	band 4.1, ezrin, radixin, moesin
FRNK	FAK-related nonkinase
HF/HC	high fat/high cholesterol
ICAM-1	intercellular adhesion molecule-1
IL-1β	interleukin-1β
KD	kinase-dead
KO	knockout
LDL	low-density lipoprotein
LDLR	low-density lipoprotein receptor
LVH	left ventricular hypertrophy
MMP	matrix metalloproteinase
oxLDL	oxidized low-density lipoprotein
PAH	pulmonary arterial hypertension
PDGF	platelet derived growth factor
PASMC	pulmonary artery smooth muscle cell
PE	phenylephrine
Pyk2	proline-rich tyrosine kinase 2
siRNA	small interfering RNA
Skp2	S-phase kinase-associated protein 2
TNF-α	tumor necrosis factor-α
VCAM-1	vascular cell adhesion molecule-1
VEGFR2	vascular endothelial growth factor receptor 2
VSMC	vascular smooth muscle cell
WT	wild-type
